# Disentangling the facilitation effect of emoji in vocabulary recognition: experimental evidence from semantic matching tasks

**DOI:** 10.3389/fpsyg.2025.1629078

**Published:** 2025-10-29

**Authors:** Yin Li, Zilong Zhong

**Affiliations:** ^1^Nanyang Institute of Technology, Nanyang, China; ^2^Beijing Foreign Studies University, Beijing, China

**Keywords:** emoji, vocabulary recognition, second language learning, multimodal learning, semantic processing

## Abstract

**Background:**

In the digital age, visual symbols such as emojis have become integral to everyday communication. Despite their ubiquity, the cognitive and educational functions of emojis remain underexplored, particularly in the context of second language (L2) learning.

**Objectives:**

This study aims to examine the potential of emojis as multimodal tools for enhancing vocabulary recognition in L2 learners. Specifically, it investigates whether emojis support more accurate and efficient semantic processing compared to traditional verbal translation methods.

**Methods:**

A 2 (text type: emoji–Chinese vs. English–Chinese) × 2 (task type: match vs. mismatch) within-subjects experimental design was employed. Thirty English-speaking learners of Chinese participated and completed a series of semantic matching tasks. Reaction times and accuracy were recorded to assess cognitive processing under each condition.

**Results:**

Participants demonstrated significantly faster response times and higher accuracy in the emoji–Chinese match condition compared to the English–Chinese condition, especially when semantic congruency was present. However, the facilitative effect of emojis was reduced in mismatch conditions, suggesting a context-dependent influence of visual symbols on learning outcomes.

**Conclusion:**

These findings highlight the potential of emojis as semiotic and cognitive resources in L2 vocabulary learning. The study underscores the importance of context-sensitive integration of visual media in instructional design, offering practical implications for enhancing language learning through multimodal approaches. The findings suggest that emoji can facilitate vocabulary recognition. However, the current results should be interpreted as evidence that emoji facilitate learners’ performance in semantic matching tasks, rather than evidence of long-term vocabulary acquisition. Future research should incorporate delayed post-tests and retention measures to examine whether emoji support durable learning outcomes.

## 1 Introduction

In the contemporary digital era, communication is increasingly shaped by multimodal resources, with visual elements such as emojis, GIFs, stickers, and memes becoming integral to digital discourse (Zappavigna and Logi, 2024; Zhong, 2024). These visual components are no longer mere peripheral decorations to text-based messages but have emerged as central semiotic resources that influence how individuals express meaning, emotion, and intention in digital contexts. The proliferation of smartphones, social media platforms, and instant messaging apps has accelerated this “visual turn” in communication, reshaping language practices both inside and outside the classroom (Highfield and Leaver, 2016; Jiang and Hafner, 2024). Emojis have become a ubiquitous and standardized means of conveying meaning, enabling people to communicate emotions, actions, and ideas across cultural and linguistic boundaries.

The significance of studying emojis in language learning has become even more apparent as they increasingly play a central role in modern communication, especially in digital environments (Chawla-Duggan, 2024; Zhong, 2025a). Their widespread use enhances engagement and helps bridge cultural and linguistic gaps, making them a promising tool for language acquisition. The ability of emojis to convey emotional and contextual information visually can significantly enrich learners’ experiences, providing an additional layer of meaning that may support more effective vocabulary learning. Recent research underscores the growing relevance of visual elements like emojis in educational settings, particularly in the context of multimodal learning environments that leverage both verbal and nonverbal cues to support cognitive and emotional engagement (Alshaya, 2025).

Among visual resources, emojis stand out due to their ubiquity, standardization, and accessibility. Originally designed as simple graphic symbols to convey emotions or actions, emojis have evolved into a translingual mode of communication used across cultures and age groups (Danesi, 2017; Kerslake and Wegerif, 2017). Their visual immediacy, emotional expressiveness, and standardized form make them highly suitable for use in pedagogical contexts, especially where learners face linguistic or cognitive barriers. Emojis do not merely “decorate” discourse; rather, they function as semiotic and cognitive tools capable of compressing complex emotional and contextual information into highly interpretable visual forms (Bahari et al., 2023). Moreover, their integration into educational materials facilitates the bridging of cognitive load gaps, supporting learners in navigating complex vocabulary and enhancing retention.

Understanding their functional role in learning environments is critical for second language (L2) education, where learners face challenges in vocabulary acquisition, semantic processing, and cross-modal integration. This research is relevant as it investigates the potential benefits of emojis in enhancing L2 learners’ vocabulary retention, bridging linguistic gaps, and supporting cognitive functions involved in language learning. Cognitive Load Theory (Sweller, 1994), Multimedia Learning Theory (Mayer, 2002), and Dual Coding Theory (Paivio, 1990) collectively offer a robust framework for understanding how emojis may reduce cognitive load, provide multimodal input, and strengthen memory through dual-channel processing.

The cognitive and pedagogical value of integrating emojis into L2 instruction can be theorized through multiple frameworks. First, dual coding theory (Paivio, 1990, 1991; Paivio and Desrochers, 1980) posits that information presented in both verbal and visual formats is more easily retained and retrieved. In language learning, presenting new vocabulary alongside meaningful images or emojis can help form stronger memory traces via verbal and nonverbal channels (Mayer, 2002). This dual representation is especially beneficial for novice learners, who often struggle with abstract word meanings and unfamiliar phonological structures (Han et al., 2023; Nation, 2001). However, it is also crucial to consider other theories such as cognitive load theory and multimedia learning theory, which highlight the importance of balancing cognitive resources and structuring multimodal input to avoid overwhelming learners. Incorporating these theories into the study of emojis in language learning allows for a more nuanced understanding of how visual cues can support cognitive processes while minimizing extraneous cognitive load. Second, theories of cognitive load (Sweller, 1994; Bahari et al., 2023) emphasize the importance of instructional design in reducing unnecessary mental effort. By acting as intuitive semantic cues, emojis can potentially reduce extraneous load and enhance the efficiency of processing during vocabulary learning. Third, multimodal learning theory suggests that meaningful integration of text, image, and affect can facilitate more embodied and holistic learning experiences, aligning with current calls to redesign language education for digital contexts (Jiang and Hafner, 2024; Gay, 2018). Together, these frameworks help to explain why emojis, as multimodal signals, can offer distinct advantages in language learning environments.

These theoretical insights resonate with ongoing innovations in Computer-Assisted Language Learning (CALL). With the rise of mobile-assisted and gamified platforms, there is a growing emphasis on multimodal input, personalization, and affectively engaging content (Buendgens-Kosten and Elsner, 2018; McCallum and Tafazoli, 2025; Stockwell, 2012; Zhong, 2025b). Prior studies have demonstrated the benefits of machine translation tools (Garcia and Pena, 2011), automated feedback systems (Wang et al., 2024), and spaced repetition algorithms (Chukharev-Hudilainen and Klepikova, 2016) in optimizing vocabulary learning. Despite these advancements, empirical studies explicitly examining the impact of emojis on vocabulary processing remain scarce, with most existing studies focusing primarily on the socio-pragmatic functions of emojis in digital discourse (Lo, 2008; Derks et al., 2008). Recent studies (Alshaya, 2025; Moffitt et al., 2021) have explored how emojis influence engagement and perceptions in educational contexts, providing a backdrop for examining their role in L2 learning.

This research aims to fill that gap by investigating the role of emojis in facilitating semantic processing in L2 vocabulary tasks. Specifically, it examines whether integrating emoji as a semantic scaffold can enhance performance compared to traditional verbal translations (e.g., English–Chinese). It also considers whether these effects are modulated by semantic congruency, that is, whether the benefit of emoji varies between matched and mismatched conditions. The research hypotheses are as follows: (1) The use of emojis will facilitate more accurate semantic processing of vocabulary in L2 learners compared to verbal translations. (2) Emoji use will enhance performance more significantly in semantically congruent conditions than in mismatched conditions. These research questions are particularly relevant in bilingual and multilingual learning environments, where learners often face difficulties managing cross-linguistic semantic interference and working memory demands (Han et al., 2024; Schwartz and Kroll, 2006).

Acknowledging the complexity of using emojis in educational contexts, it is important to also consider the potential negative effects. These may include the risk of oversimplification, misinterpretation, and distraction. For instance, emojis may oversimplify or distort nuanced meanings of words, potentially leading to inaccurate interpretations. Moreover, excessive reliance on emojis might cause learners to focus more on visual cues than on the linguistic content, leading to distraction. Therefore, careful integration of emojis into structured educational tasks and contexts is essential to ensure that they enhance rather than hinder the learning process. This is particularly important when considering cultural differences in emoji interpretation, as emojis may hold different connotations depending on cultural backgrounds (Sun et al., 2022).

By adopting a multimodal semantic matching paradigm, this study seeks to provide empirical evidence for how emojis influence real-time language processing in L2 learners. In doing so, it contributes to a growing body of research exploring how digital and visual media can be meaningfully integrated into language learning environments. The findings not only inform theoretical models of multimodal and bilingual cognition but also offer practical guidance for designing innovative, culturally responsive CALL interventions that align with 21st-century digital literacies (Gay, 2018; Wang and Li, 2023). This study also contributes to the broader conversation on integrating artificial intelligence and emoji systems in educational technology (Alshaya, 2025; Chen et al., 2022), highlighting how AI-driven tools could support personalized, emotion-aware learning experiences.

## 2 Research method

### 2.1 Experimental design

A 2 (text type: emoji–Chinese vs. English–Chinese) × 2 (task type: match vs. mismatch) within-subjects factorial design was employed in the experiment. The independent variables were text type and task type, while the dependent variables were participants’ response times and accuracy in the semantic matching tasks. In each trial, participants were presented with a combination of either emoji or English text alongside Chinese text, and their task was to determine whether the texts were semantically matched or mismatched. The design allowed for the examination of how different text types (emoji–Chinese vs. English–Chinese) and task types (match vs. mismatch) influenced the efficiency and accuracy of vocabulary processing.

### 2.2 Sample size estimation

To determine the required sample size for this experiment, *a priori* power analysis was conducted using G*Power 3.1.9 (Faul et al., 2007). The analysis was based on the following parameters: effect size *f* = 0.25, *α* error probability = 0.05, power (1-*β* error probability) = 0.80, with one group and four measurements per participant. The correlation among repeated measures was set at 0.5, and the nonsphericity correction was set to *ε* = 1. Based on these input parameters, the required total sample size was calculated to be 24 participants. The analysis also yielded a critical *F* value of 2.737, with numerator degrees of freedom (*df*) = 3 and denominator *df* = 69. The actual power of the analysis was 0.82, indicating a sufficient sample size for detecting the effects in the experimental design.

### 2.3 Participants

Based on the result of the power analysis, a total of 30 international students (13 males, 17 females) from a university in Beijing participated in the experiment. Participants’ ages ranged from 18 to 20 years, with an average age of 19.03 years (*SD* = 0.75). All participants were English learners of Chinese as a foreign language (CFL), with English as their native language (L1) and Chinese as their second language (L2). The participants had been learning Chinese for 1 to 2 years, with an average learning duration of 1.40 years (*SD* = 0.49). Prior to the experiment, participants completed a language learning history questionnaire, which included self-assessments of their proficiency in L1 and L2 on a 7-point scale (with “7” indicating very proficient and “1” indicating very unskilled). The average self-assessment for L1 proficiency was 6.93 (*SD* = 0.11), while the average self-assessment for L2 proficiency was 2.65 (*SD* = 0.18). A t-test comparing the self-reported proficiency in L1 and L2 revealed a significant difference (*p* < 0.001). In addition, participants filled out a questionnaire on emoji usage, which asked them to rate their frequency of emoji usage on social media on a 7-point scale (with “7” indicating frequent use and “1” indicating no use). The results showed no significant difference in emoji usage frequency among participants (*p* > 0.050), which helped to control for any potential biases related to varying levels of emoji familiarity prior to the experiment. All participants had no history of brain injuries or psychiatric disorders, had normal or corrected-to-normal vision, and were not colorblind. Before the experiment, participants signed a paper-based informed consent form, and they received compensation at the end of the experiment.

### 2.4 Experimental materials

The materials used in this study consisted of 20 sets of Chinese–English emotional words (e.g., “开心,” “happy”) and their corresponding emoji symbols (e.g., “
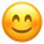
”), comprising 10 sets of positive emotional words and 10 sets of negative emotional words. To control potential confounding variables, the two categories of words were matched on arousal, pleasantness, and abstractness using a 7-point Likert scale (1 = very low, 7 = very high). A t-test revealed no significant differences between positive and negative words in arousal and abstractness (*p* > 0.050), while the pleasantness of positive words was significantly higher than that of negative words (*p* < 0.050). To ensure comparable usage frequency between positive and negative words, their frequencies in Chinese and English were matched using the Beijing Language and Culture University Chinese Corpus (BCC), and the Corpus of Contemporary American English (COCA) respectively. As a result, no significant differences were found in their usage frequencies (*p* > 0.050), minimizing the potential influence of memory-related processing fluency. In this study, we chose the English–Chinese language pair as a baseline condition for comparison with the emoji–Chinese condition. The primary aim of the study was to investigate whether emoji can facilitate vocabulary recognition, and English–Chinese provided a common reference point for this comparison. Participants were asked to perform a semantic matching task by matching the presented stimuli [either emoji–Chinese (e.g., “
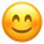
–开心”) or English–Chinese (e.g., “happy–开心”)]. The emoji symbols used in this experiment were all sourced from the open emoji library website^1^ and were designed by Apple Inc. During the experiment, both stimulus types (emoji–Chinese and English–Chinese) were presented in equal numbers to ensure a balanced representation of the languages and to allow for robust analysis across different experimental conditions. This study focused on a micro-level vocabulary recognition task to investigate the specific effects of emoji on vocabulary learning, with the number of word sets chosen to maintain experimental control and reduce cognitive overload. The decision to use only emotional words was made to control for potential confounding variables and ensure the validity of the results. Emotional words tend to evoke stronger reactions, which allowed us to isolate the specific impact of emoji on vocabulary recognition.

### 2.5 Experimental procedure

**Figure 1:**
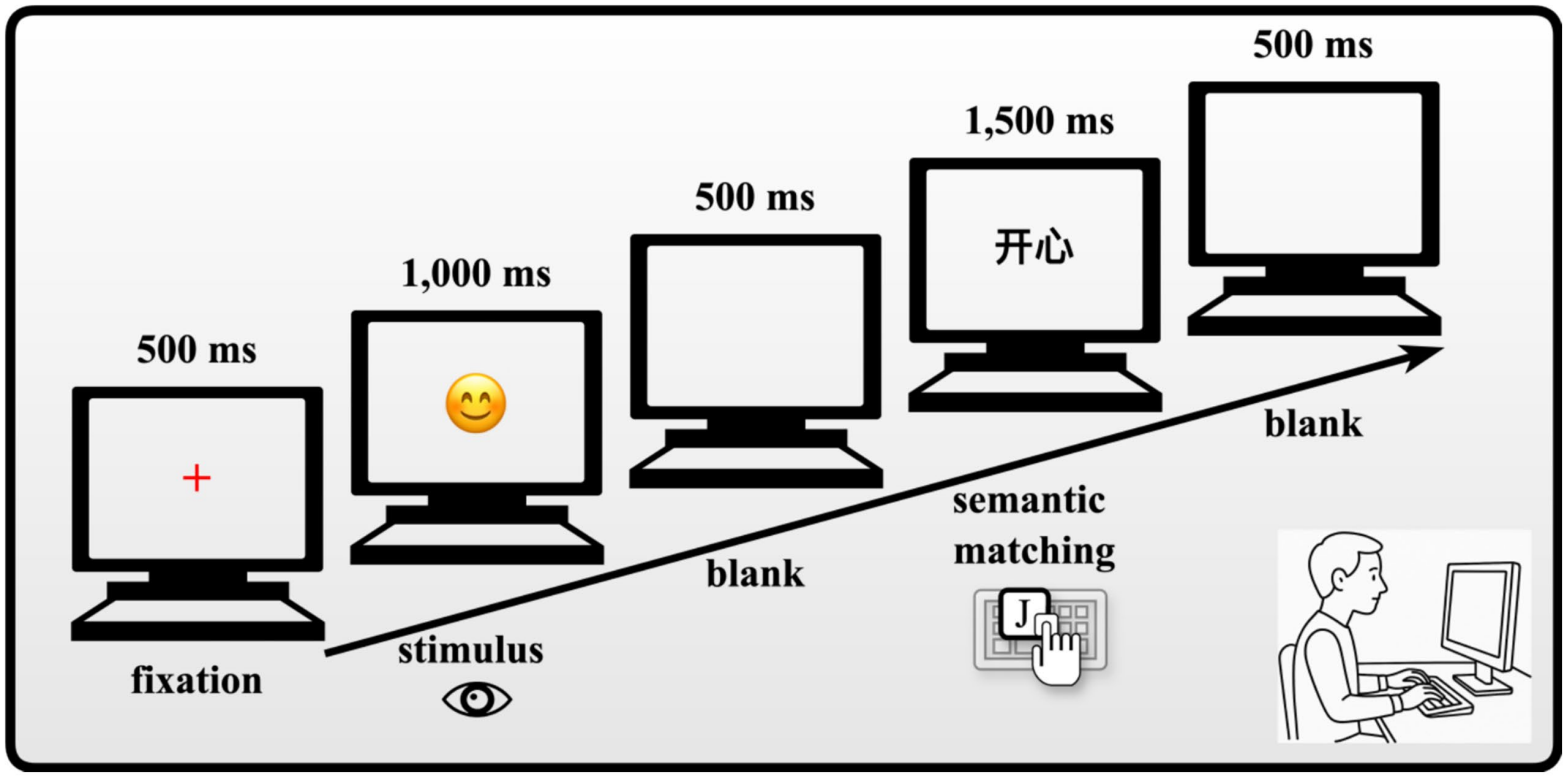
Experimental procedure.

The experiment was programmed using E-Prime 3.0, which was used for presenting the materials and collecting data. Prior to the formal experiment, participants completed a practice session consisting of 12 trials, which could be repeated as needed until the participants were familiar with the experimental procedure. Once they were ready, they pressed the “q” key to begin the formal experiment. Each trial began with a 500 ms fixation cross (“+”) presented in red, followed by the presentation of either an English emotional word or an emoji for 1,000 ms. After a 500 ms blank screen, a Chinese emotional word appeared, and participants were required to semantically match the two stimuli (either emoji–Chinese or English–Chinese). If the two stimuli matched semantically (e.g., “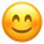 –开心”), participants were instructed to press the “J” key; if they did not match (e.g., “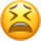 –开心”), they pressed the “F” key. If no response was made within 1,500 ms, the stimuli disappeared. There was a 500 ms interval between each trial (see Figure 1 for the experimental timeline). The computer automatically recorded the response times and accuracy. The formal experiment consisted of 3 blocks, with each block containing 40 trials, for a total of 120 trials. The number of occurrences of each type of text (emoji–Chinese, English–Chinese) and task (match, mismatch) was balanced across the blocks. Between blocks, participants were free to choose whether to take a break and how long to rest, ensuring they remained focused throughout the experiment. Experimental stimuli were presented in pseudo-randomized sequences, and the order of conditions was systematically counterbalanced across participants to minimize potential order effects. The presentation order of the stimuli was unpredictable for the participants, and the programming prevented direct repetition of stimuli, thus minimizing potential interference from prior exposure to the same stimulus. This approach helped maintain the integrity and validity of the experimental design, enhancing the reliability of our findings.

### 2.6 Data analysis

The data analysis for this study was conducted using R software (R Core Team, 2023) with the lme4 package (Bates et al., 2015) to fit linear mixed-effects models (LMMs) in order to assess the effects of different experimental conditions on participants’ reaction times and accuracy.

In the data preprocessing phase, reaction time data were screened to remove extreme values. Specifically, trials with reaction times below 200 ms, as well as trials with reaction times outside ±2.5 standard deviations from the mean, were excluded to minimize the impact of outliers. The accuracy data, being binary in nature (correct = 1, incorrect = 0), were modeled using a binomial distribution, as required by the nature of the dependent variable. To reduce the potential impact of multicollinearity on model estimation, all predictor variables (text type and task type) were centered prior to modeling.

To fit the best model, we began with a comprehensive model containing the maximal random effects structure, including all potential random intercepts and random slopes. If the model failed to converge or exhibited a singular fit, we gradually simplified the random effects structure until convergence was achieved. The final model for accuracy was specified as follows: accuracy∼text_type×task_type+(1∣subject) + (1 + task_type∣item). In this model, the fixed effects include text type, task type, and their interaction. This structure was used to evaluate the significant contributions of these factors and their interactions to accuracy. The random effects part of the model accounts for the random intercept for subjects to control for individual baseline differences, and random intercept and random slope for task type at the item level to account for differences in how the experimental materials affected the task performance. The final model for reaction time was specified as follows: rt. ∼ text_type×task_type+(1 + text_type∣subject) + (1∣item). This model included the same fixed effects as the accuracy model (text type, task type, and their interaction). The random effects structure allowed for random intercepts and random slopes for text type at the subject level, enabling the model to account for individual differences in how reaction time varied based on text type. Additionally, a random intercept for items was included to control for baseline differences between experimental materials. Both models were fitted using maximum likelihood estimation (MLE). The binomial accuracy data were analyzed using the glmer() function, while the normal reaction time data were analyzed using the lmer() function in the lme4 package.

To further explore the significant main effects and interactions, we used the emmeans package (Lenth, 2020) to calculate marginal means. Pairwise comparisons were then performed based on the marginal means to examine specific patterns of significant effects.

## 3 Results

**Figure 2:**
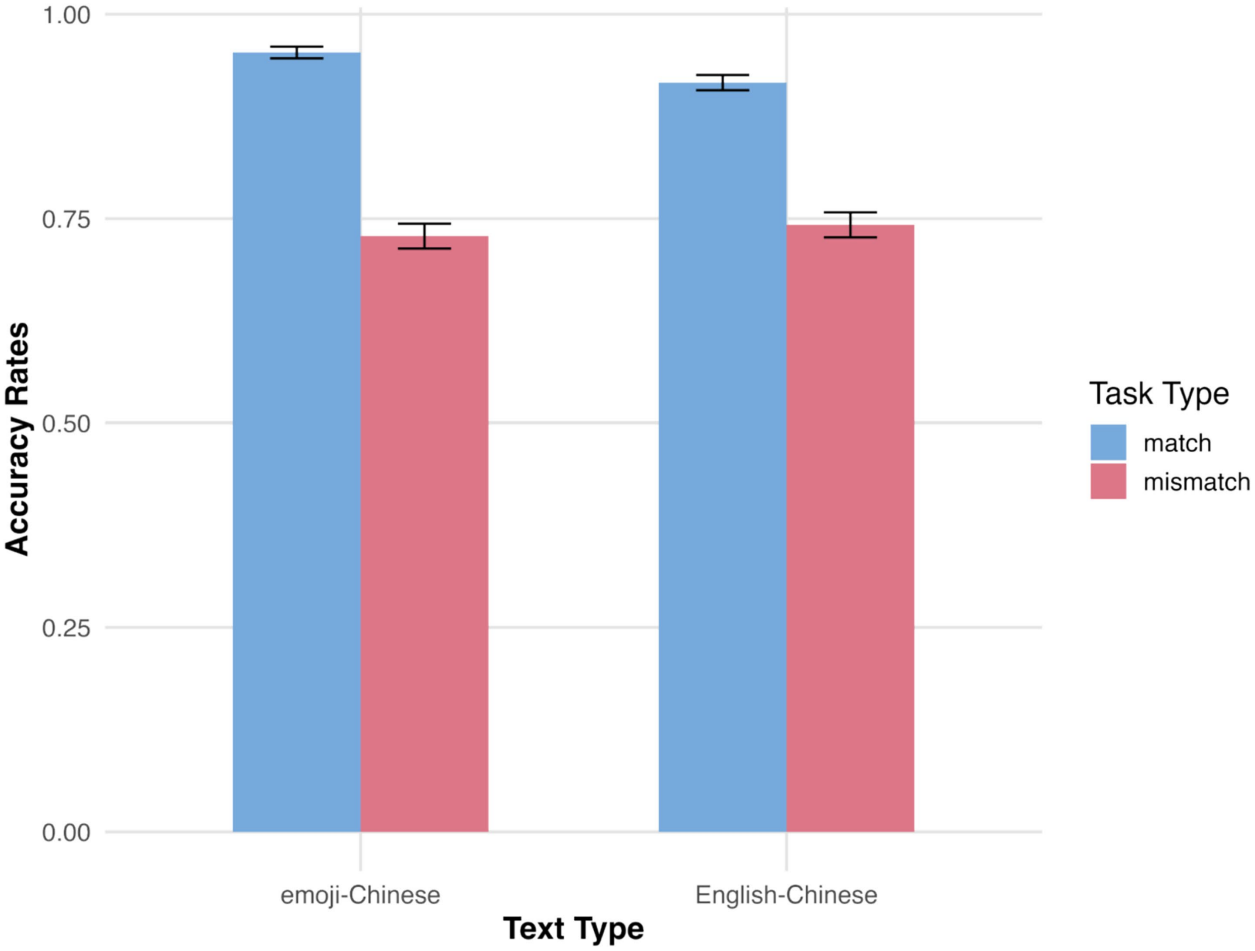
Average accuracy rates in semantic matching tasks.

**Figure 3:**
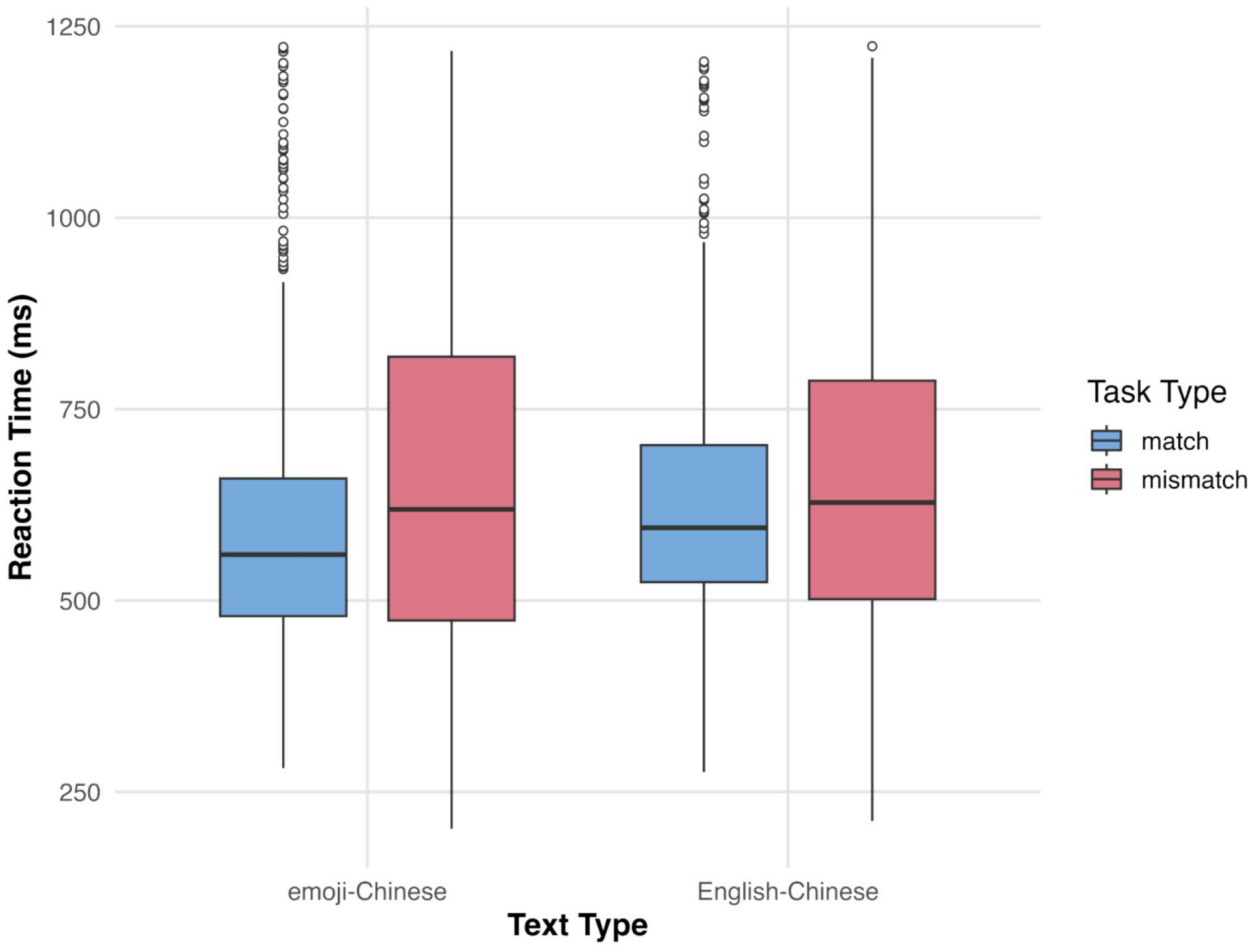
Average reaction times in semantic matching tasks.

The dependent variables in this study were participants’ semantic matching accuracy and reaction times. Accuracy analysis included both correct and incorrect responses, while reaction time analysis was restricted to correct responses. Statistical analyses of both accuracy and reaction time data are presented below. The average accuracy and reaction times for participants’ semantic matching are shown in Figures 2, 3. In addition to reporting statistical significance, we also provide effect sizes to facilitate interpretation of the practical significance of our findings. For the accuracy data analyzed with generalized linear mixed model (GLMM), we report Cohen’s d, as the model uses a binomial distribution with a logit link, making the raw coefficients difficult to interpret in a meaningful metric such as percentage accuracy. Cohen’s d, by contrast, standardizes the mean difference between conditions relative to the pooled standard deviation, allowing readers to understand the magnitude of the observed effects on a common scale. This makes it especially appropriate for categorical or proportion-based outcomes, where the direct model estimates do not convey intuitive differences in performance. For the reaction time data analyzed with LMM, we report η^2^ (eta-squared), which quantifies the proportion of total variance in the dependent variable explained by each predictor. Reaction time is a continuous variable with approximately normal distribution after transformation, and η^2^ is a natural measure of effect size for continuous outcomes analyzed with linear models. It allows readers to interpret how much of the variability in reaction times can be attributed to each experimental factor, offering an interpretable index of practical significance parallel to the familiar concept of explained variance (R^2^). Effect sizes are presented in Tables 1, 2. Intercept terms are not accompanied by effect sizes, as they reflect baseline means rather than comparative effects between conditions.

**Table 1:** Best-fitting model output for accuracy.

Indexes	Fixed effects					Random effects	
	Estimate	SE	Pr (>|z|)	95% CI	Cohen’s d	SD by subject	SD by item
Intercept (emoji–Chinese, match)	3.1552	0.2008	< 2e-16 ***	[2.7615, 3.5489]	–	0.2829	0.4871
Text type (English–Chinese)	−0.6295	0.2388	0.00839 **	[−1.0996, −0.1594]	−0.347		
Task type (mismatch)	−2.0925	0.2316	< 2e-16 ***	[−2.5476, −1.6374]	−1.154		0.9549
Text type (English–Chinese): task type (mismatch)	0.7170	0.2995	0.01667 *	[0.1293, 1.3047]	0.395		

Signif. codes: 0 “***” 0.001 “**” 0.01 “*” 0.05 “.” 0.1 “” 1.

**Table 2:** Best-fitting model output for reaction times.

Indexes	Fixed effects					Random effects	
	Estimate	SE	Pr(>|t|)	95% CI	η^2^	SD by subject	SD by item
Intercept (emoji–Chinese, match)	590.97	12.83	<2e-16 ***	[565.13, 616.81]	–	56.20	22.99
Text type (English–Chinese)	42.06	15.53	0.00926 **	[10.84, 73.28]	0.128	60.12	
Task type (mismatch)	61.40	11.57	5.4e-07 ***	[38.46, 84.34]	0.196		
Text type (English–Chinese): task type (mismatch)	−35.51	16.46	0.03301 *	[−68.07, −2.95]	0.138		

Signif. codes: 0 “***” 0.001 “**” 0.01 “*” 0.05 “.” 0.1 “” 1.

### 3.1 Semantic matching accuracy

The analysis of participants’ accuracy in the semantic matching tasks (see Table 1) revealed several significant effects. A significant main effect of text type was observed (*z* = −2.636, *p* < 0.010), indicating that participants exhibited significantly higher semantic matching accuracy for emoji–Chinese stimuli compared to English–Chinese stimuli. There was also a significant main effect of task type (*z* = −9.036, *p* < 0.001), with participants showing higher accuracy in semantic matching tasks than in semantic mismatching tasks. Moreover, the interaction between text type and task type was significant (*z* = 2.394, *p* < 0.050). Pairwise comparisons revealed that participants achieved significantly higher accuracy in the emoji–Chinese semantic matching task than in the English–Chinese semantic matching task (*z* = 2.636, *p* < 0.050), the emoji–Chinese semantic mismatching task (*z* = 9.036, *p* < 0.001), and the English–Chinese semantic mismatching task (*z* = 8.603, *p* < 0.001). Additionally, participants’ accuracy in the English–Chinese semantic matching task was significantly higher than in both the emoji–Chinese semantic mismatching task (*z* = 7.100, *p* < 0.001) and the English–Chinese semantic mismatching task (*z* = 6.621, *p* < 0.001). However, no significant difference in accuracy was found between the emoji–Chinese and English–Chinese semantic mismatching tasks (*p* > 0.050).

### 3.2 Semantic matching reaction times

The analysis of participants’ reaction times in the semantic matching tasks (see Table 2) revealed several significant effects. A significant main effect of text type was found (*t* = 2.708, *p* < 0.010), with participants responding significantly faster to emoji–Chinese stimuli than to English–Chinese stimuli. A significant main effect of task type was also observed (*t* = 5.308, *p* < 0.001), indicating that participants responded significantly faster in semantic matching tasks compared to semantic mismatching tasks. Furthermore, there was a significant interaction between text type and task type (*t* = −2.157, *p* < 0.050). Pairwise comparisons showed that participants responded significantly faster in the emoji–Chinese semantic matching task than in the emoji–Chinese semantic mismatching task (*t* = −5.307, *p* < 0.001), the English–Chinese semantic matching task (*t* = −2.707, *p* < 0.050), and the English–Chinese semantic mismatching task (*t* = −4.247, *p* < 0.001). No other comparisons reached statistical significance (*p* > 0.050).

The differences in reaction times across the conditions are meaningful in educational contexts. While statistically significant differences were observed, it is important to assess the practical significance of these differences. A reaction time difference, which was commonly observed in this study, could be considered a meaningful threshold for real-world learning applications, particularly in language education. These small but significant reaction time advantages could reflect the facilitative role of emojis in vocabulary recognition, supporting their potential application in classroom settings. In practical terms, such differences in reaction times may enhance the efficiency of learning tasks, making emoji-based approaches more engaging and cognitively efficient for learners.

## 4 Discussion

### 4.1 The role of emoji in semantic integration

The observed main effect of text type, where participants exhibited significantly higher accuracy for emoji–Chinese stimuli compared to English–Chinese stimuli, provides empirical support for Dual Coding Theory (Paivio, 1990; Paivio and Desrochers, 1980) and aligns with broader frameworks in multimodal and computer-assisted language learning (Buendgens-Kosten and Elsner, 2018; Bahari et al., 2023). According to Dual Coding Theory, verbal and nonverbal information are processed through two distinct but interconnected cognitive systems. In this context, emojis function as visual, emotionally salient signs that activate the imagery-based system, facilitating more robust semantic encoding and retrieval of the paired Chinese words. This additional representational channel may be particularly beneficial in L2 contexts where lexical representations are still developing (Han et al., 2023). However, it is important to note that the present study only tested the immediate effects of emoji use in a specific experimental context. Therefore, these findings should be interpreted with caution, as they do not necessarily extend to longer-term learning outcomes or other contexts. The results also resonate with embodied and affective models of cognition, which suggest that emotionally meaningful visuals, such as facial expressions in emojis, can engage sensorimotor systems and deepen conceptual processing (Barsalou, 2008; Niedenthal, 2007). However, further research is needed to examine whether these embodied effects persist in different learning contexts or with different learner populations.

The significant main effect of task type, with participants achieving higher accuracy in semantic match trials than mismatch trials, is consistent with predictions from semantic priming and predictive coding models of language comprehension (Federmeier, 2007; Kutas and Federmeier, 2011). Semantic congruency allows for top-down facilitation, whereby prior expectations streamline processing of upcoming input, reducing the need for complex integrative operations. Mismatching trials, by contrast, create semantic interference, requiring learners to suppress activated expectations, an effortful process especially for bilingual and multilingual learners with varying proficiency levels (Han et al., 2024). This effect further underscores the cognitive load imposed by incongruent multimodal stimuli, which require integration across visual and verbal modalities.

The observed interaction between text type and task type highlights the conditional benefits of emoji support. The highest accuracy in the emoji–Chinese match condition suggests that emojis not only serve as semantic scaffolds but may also enhance encoding through associative reinforcement when the symbolic visual aligns with the target lexical item. This effect aligns with the Affective Embodiment Hypothesis (Niedenthal, 2007), which proposes that emotional visual stimuli engage embodied simulation mechanisms, thereby strengthening semantic memory traces. Given that emojis typically express affect through highly recognizable facial features or gestures, they amplified semantic resonance during congruent trials.

However, the lack of significant advantage in the emoji–Chinese mismatch condition, relative to the English–Chinese mismatch condition, points to a crucial caveat. While emojis facilitated semantic matching in congruent conditions, they did not offer advantages in mismatched contexts. The facilitative effect of emojis appears context-dependent, exerting cognitive benefits only when the visual and verbal stimuli are semantically aligned. In incongruent conditions, the strong emotional or intuitive associations triggered by emojis may hinder processing, introducing semantic dissonance and increasing decision uncertainty. This aligns with findings in multimodal processing research, which show that incongruity between visual and verbal information imposes greater working memory demands and cross-code verification burdens (Wong and Maurer, 2021; Chukharev-Hudilainen and Klepikova, 2016).

From a second language acquisition (SLA) perspective, these findings offer nuanced insights into the cognitive mechanisms involved in vocabulary learning and comprehension. Whereas written L1 translations (e.g., English words) require lexical retrieval and syntactic parsing, processes subject to interference and code-switching costs (Lu et al., 2019; Schwartz and Kroll, 2006), emojis bypass linguistic decoding, allowing learners to directly access semantic content. This may reduce extraneous cognitive load (Sweller, 1994; Bahari et al., 2023) and support germane load, particularly when paired with congruent verbal input. However, it is essential to acknowledge that these conclusions are based on immediate effects observed in the specific context of this study. The generalizability of these findings across different L2 learners, language pairs, and types of vocabulary tasks remains uncertain, and further research is necessary to explore these dimensions.

These results also have important pedagogical implications. In line with emerging innovations in digital multimodal composition and game-based CALL environments (Stockwell, 2012; Deterding et al., 2011; Wang and Li, 2023), the strategic use of emojis could support more engaging and effective L2 instruction. However, the study emphasizes that the implications for pedagogy are preliminary. Given that the study only tested immediate task performance in one specific context, these implications should be considered with caution. Multimodal supports such as emojis must be applied contextually and carefully, as incongruent emoji usage, rather than being neutral, may actively impede comprehension if the semantic message contradicts the intended meaning of the target language. Therefore, emoji integration should be systematically aligned with learning goals and tested for congruency effects before broad instructional implementation.

Finally, this study contributes to a growing body of research advocating for culturally responsive, technology-enhanced language teaching (Gay, 2018; Garcia and Pena, 2011; McCallum and Tafazoli, 2025). It highlights the value of designing vocabulary tasks that reflect authentic digital communication practices, including emoji use, while also ensuring that instructional strategies are cognitively grounded and empirically validated. Future research should explore how these effects generalize across different learner populations, language pairs, and task types, and how emoji–based interventions can be optimized through adaptive or personalized learning technologies (Wang et al., 2024).

### 4.2 Visual symbols as cognitive shortcuts

The reaction time results offer compelling evidence for the processing advantage of emoji in L2 vocabulary tasks, particularly under conditions of semantic congruency. Participants responded significantly faster to emoji–Chinese pairs than to English–Chinese pairs, indicating that visual symbols, particularly affect-laden and universally recognizable emojis, can enhance lexical access and semantic integration more efficiently than alphabetic translation equivalents. However, it is important to note that these conclusions are based on the immediate effects observed within the specific experimental context of this study. Further research is needed to confirm whether these effects extend beyond the tested conditions. This advantage supports Dual Coding Theory (Paivio, 1990, 1991), which posits that when learners are presented with both verbal and visual inputs, the information is processed through two complementary cognitive systems, leading to more efficient memory retrieval and faster decision-making. The task-specific effects observed in this study are promising but should be interpreted with caution, as they are not generalized across other types of learning contexts or vocabulary tasks.

The affective and iconic nature of emojis contributed to holistic and rapid recognition of emotional meaning, facilitating quicker mapping onto the target L2 (Chinese) vocabulary. Such processing aligns with affective priming models (Hermans et al., 1994), in which emotionally congruent primes (in this case, emojis) speed up the processing of semantically related targets due to pre-activated associative networks.

From a semiotic perspective, the reaction time benefits of emoji reflect their status as iconic signs (Peirce, 1935), in which meaning is directly perceived through visual resemblance. This contrasts with alphabetic English words, which operate via symbolic codes that require more abstract decoding. For CFL learners, often navigating complex cross-linguistic mappings between a dominant L1 and a less familiar L2, the cognitive detour involved in lexical translation can slow processing (Lu et al., 2019; Schwartz and Kroll, 2006). Emojis offer an intuitive semantic shortcut, bypassing phonological and syntactic processing layers and enabling learners to access meaning through direct visual-emotional channels (Han et al., 2023).

The significant main effect of task type, wherein responses were faster in match trials than mismatch trials, further demonstrates the role of predictive semantic processing. In match trials, learners benefited from top-down expectancy mechanisms (Clark, 2013), where congruent visual primes facilitated anticipatory activation of semantically related L2 vocabulary. This was especially true in emoji conditions, where emotionally clear visuals may have prepared participants for affectively congruent Chinese terms. In mismatch trials, this expectation was violated, necessitating cognitive inhibition, semantic reevaluation, and longer response times, consistent with theories of conflict monitoring and semantic mismatch costs (Kutas and Federmeier, 2011; Bahari et al., 2023).

Crucially, the interaction between text type and task type pinpoints the specific context in which emoji use conferred the greatest advantage: the emoji–Chinese match condition. In this condition, reaction times were significantly faster than in all other conditions, supporting models of intersemiotic complementarity (Bateman, 2014), which argue that multimodal messages enhance comprehension when the modes (e.g., visual and verbal) share congruent meaning. The universality of emoji facial expressions and the affective resonance they carry may have provided redundant semantic cues that reinforced the target vocabulary and expedited retrieval.

However, this advantage was not unconditional. In mismatch conditions, the reaction time benefit of emoji disappeared. This finding underscores that the facilitative effects of emojis are contingent on semantic congruency. When an emoji evokes a strong but mismatched emotional meaning [e.g., a smiling face followed by the word “难过 (sad)”], learners must override pre-activated semantic expectations, which imposes additional cognitive demand. This pattern mirrors earlier findings in bilingual processing, where semantic incongruity across modalities or languages increases processing difficulty and impairs efficiency (Wong and Maurer, 2021; Schwartz and Kroll, 2006).

These results carry important pedagogical implications for second language instruction in digital and multilingual contexts. The reaction time advantage of emojis in congruent conditions highlights their potential as multimodal learning aids that can reduce cognitive load (Sweller, 1994), increase learner engagement (Deterding et al., 2011), and facilitate faster access to L2 meanings, especially for abstract or affective vocabulary. However, the study emphasizes that the implications for pedagogy are preliminary. Given that this study only tested the immediate effects on task performance in a single context, these suggestions should be considered with caution. The performance drop in incongruent trials emphasizes the need for careful instructional design. Emojis should be integrated strategically and semantically aligned with target vocabulary to avoid misleading cues and semantic confusion. These findings support broader efforts to foster visual literacy and semiotic awareness in CALL and language pedagogy (Jiang and Hafner, 2024; Gay, 2018), helping learners become more adept at interpreting and regulating multimodal inputs.

In sum, the reaction time data clarify a critical boundary condition: emoji facilitate faster vocabulary processing when their visual and emotional content aligns with the intended meaning. In such cases, they act as efficient, emotionally resonant visual-semantic shortcuts. When misaligned, they may create semantic noise that disrupts processing. These insights contribute to the broader aims of this study, clarifying when and how emojis enhance language learning, and underscore the value of theoretically grounded, context-sensitive, and learner-centered design principles for integrating visual tools in language education. Further research is needed to explore how these effects generalize across different learner populations, languages, and instructional settings.

### 4.3 Limitations and considerations for generalizability

While the findings of this study provide insights into the role of emojis in vocabulary recognition, there are several limitations to consider, which may affect the generalizability of the results.

First, the use of a single language pair (English–Chinese) as the baseline condition limits the generalizability of the findings to other language pairs. Although English–Chinese provides a common reference point for comparison, it is important to recognize that the cognitive processes involved in learning different language pairs may vary significantly. Future research should explore a wider range of language pairs to assess whether the observed effects of emoji use are applicable across various linguistic contexts. This would help determine the broader relevance of the findings to learners of other languages and further test the robustness of emoji-based interventions.

Second, the restricted focus on emotional words in this study, while helpful for isolating the specific impact of emoji on vocabulary recognition, limits the scope of vocabulary learning examined. Emotional words tend to evoke stronger emotional reactions, which facilitated the effects observed in this experiment. We therefore caution that our findings should be interpreted as specific to emotional vocabulary, where emojis may have stronger associations, rather than generalizable to all types of vocabulary. Future research should expand the scope to include more diverse word categories, such as neutral or abstract terms, to test whether the effects of emoji use extend beyond emotional content.

Third, the absence of testing for long-term retention is another limitation. This study focused on the immediate effects of emoji use in vocabulary recognition tasks, but it did not assess whether these effects were sustained over time. Thus, the present findings should be understood as evidence that emojis can facilitate learners’ performance in immediate semantic matching tasks, rather than evidence of durable vocabulary acquisition. Future studies should incorporate delayed post-tests or retention measures to evaluate whether the observed benefits of emoji use persist over time and contribute to genuine long-term learning.

Fourth, the study also utilized a relatively small number of word sets, which may limit the robustness and generalizability of the findings. While this micro-level vocabulary recognition task provided insights into the immediate effects of emoji use, the small sample size of word sets may not fully capture the complexity of vocabulary learning. Future studies could include larger sets of words to test the reliability of these results and to extend the applicability of the findings to more comprehensive vocabulary learning tasks.

Fifth, we acknowledge the small sample size and the focus on immediate effects as potential limitations in generalizing the results. A larger sample size and the inclusion of longitudinal measures would allow for a more accurate assessment of the broader impact of emoji use on language learning, as well as the long-term effects on retention and comprehension.

Sixth, it is important to consider the potential problems associated with the use of emojis in educational settings. While emojis may facilitate learning when used appropriately, there is a risk that they could oversimplify or misinterpret meanings, or even distract learners from the core content if not carefully integrated. Emojis should be aligned with the learning objectives and contextually relevant to the material. Therefore, educators must be thoughtful in selecting when and how to incorporate emojis into the curriculum, ensuring that they enhance rather than hinder the learning process.

Finally, we acknowledge the cultural context in emoji interpretation. Emojis are not universally understood in the same way across different cultures, and this variability in interpretation could influence their effectiveness as learning tools. Future research should explore how cultural differences in emoji interpretation may affect their use in educational settings, especially in diverse or multilingual classrooms.

## 5 Conclusion

This study set out to disentangle the benefit effect of emoji in vocabulary recognition by examining participants’ performance in semantic matching tasks involving emoji–Chinese and English–Chinese stimulus pairs. Drawing on cognitive, semiotic, and psycholinguistic frameworks, the results clearly demonstrated that emojis enhance both the accuracy and speed of semantic processing compared to traditional verbal (English) translations, but critically, these benefits are context-dependent. Participants exhibited significantly higher accuracy and faster reaction times in emoji–Chinese match conditions than in all other conditions, highlighting the powerful role of semantic congruency between visual and verbal inputs. These findings are specific to the conditions tested in this study and pertain only to the immediate effects observed in the semantic matching task. Further research is needed to assess whether these effects extend to other tasks, vocabulary types, or learning contexts.

The findings contribute to the growing field of multimodal language learning research by providing experimental evidence that emojis, as iconic visual signs, can serve as effective cognitive scaffolds for vocabulary acquisition. By bypassing complex phonological decoding and tapping into intuitive, affective, and embodied routes to meaning, emojis offer learners an efficient and emotionally resonant pathway to accessing L2 vocabulary. However, the study underscores that these benefits are contingent on the alignment between visual and verbal stimuli, as the facilitative effects of emojis diminish when there is semantic incongruence. When visual cues conflict with verbal content, the facilitative effect of emoji diminishes and may even introduce cognitive interference. This finding emphasizes the need for careful integration of emojis in educational design, where the congruency between emoji and verbal cues must be ensured.

These results advance theoretical understanding of how visual and linguistic modalities interact during second language processing and suggest practical guidelines for the context-sensitive integration of visual media in educational design. The study makes a novel contribution by bridging visual semiotics, cognitive psychology, and psycholinguistics to systematically explore emoji’s role in semantic learning processes. While previous research has often focused on emoji as social–emotional markers, this study positions them as active semiotic resources that can significantly shape cognitive outcomes in language learning environments.

Future research could extend these findings in several important ways. First, longitudinal studies are needed to examine whether emoji-supported vocabulary learning leads to durable, long-term retention and transfer effects. Second, research should explore individual differences, such as emoji literacy, cultural familiarity with emoji representations, or levels of second language proficiency, to better understand for whom and under what conditions emoji facilitate or hinder learning. Third, future investigations might expand the range of semantic domains beyond emotional vocabulary to include abstract, technical, or culturally specific terms, thus testing the limits of emoji’s visual affordances. Finally, neurocognitive methods such as event-related potentials (ERP) could be employed to map the real-time neural dynamics underlying emoji-augmented semantic processing, providing even deeper insights into the cognitive mechanisms at work.

In conclusion, the present study highlights the promise and the complexity of integrating visual symbols like emoji into vocabulary learning and teaching. As digital communication continues to evolve toward increasingly multimodal forms, understanding the cognitive and educational implications of visual language will be vital for designing effective learning environments that harness the full potential of both verbal and visual meaning-making systems. However, the implications of this study are limited to the specific experimental conditions tested, and future research is needed to explore the broader applicability of these findings.

## Data Availability

The raw data supporting the conclusions of this article will be made available by the authors, without undue reservation.

## Footnotes

^1^ https://www.emojiall.com
